# A randomized placebo-controlled clinical study of *nab*-paclitaxel as second-line chemotherapy for patients with advanced non-small cell lung cancer in China

**DOI:** 10.1042/BSR20170020

**Published:** 2017-07-27

**Authors:** Yueming Wu, Jiang Feng, Weiwei Hu, Qingquan Luo

**Affiliations:** 1 Department of Thoracic Surgery, The People’s Hospital of Dongyang City, 60 Wuning West Road, Dongyang City, Zhejiang Province 322100, China; 2 Department of Thoracic Surgery, Shanghai Chest Hospital, Shanghai JiaoTong Universty, Shanghai 200030, China

**Keywords:** NSCLC, nab-paclitaxel, OS, PFS, second-line

## Abstract

We performed a randomized and placebo-controlled clinical study to investigate whether *nab*-paclitaxel can improve survival in patients with advanced non-small cell lung cancer (NSCLC) after unsuccessful first-line chemotherapy. Patients with stages III to IV advanced NSCLC after first-line platinum-based chemotherapy failure were randomly assigned in a 1:1 ratio to receive second-line treatment of *nab*-paclitaxel or placebo. Ninety two eligible patients were enrolled in the study. The median progression-free survival (PFS) was 4.6 months (95% confidence interval (CI): 3.4–6.7 months) for *nab*-paclitaxel, compared with 2.0 months (95% CI: 0.9–4.3 months) for placebo, representing a 56% reduction in disease progression (hazard ratio: 0.62; 95% CI: 0.33–0.81; *P*<0.001). The median overall survival (OS) was 6.3 months (95% CI: 3.9–8.2 months) for *nab*-paclitaxel, compared with 4.9 months (95% CI: 2.1–5.9 months) for placebo, representing a 22% reduction in disease progression (hazard ratio: 0.71; 95% CI: 0.33–0.85; *P*<0.001). Adverse events (AEs) were also observed for *nab*-paclitaxel. *Nab*-paclitaxel can improve survival in patients with advanced NSCLC after unsuccessful first-line chemotherapy.

## Introduction

Lung cancer is the most frequently diagnosed cancer and the leading cause of cancer deaths in China and worldwide [1]. An estimated 1.8 million new lung cancer cases occurred in 2012, accounting for approximately 13% of total cancer diagnoses [2]. More than 80% of the lung cancer cases are non-small cell lung cancer (NSCLC) [3]. Although the 5-year survival rate for patients with localized NSCLC is more than 50%, approximately 57% patients have advanced/metastatic NSCLC at the time of diagnosis with only 5% survival rate [4]. The standard first-line therapy for advanced NSCLC without targetable genetic alterations is a platinum-based doublet including third-generation cytotoxic agents (e.g. cisplatin plus gemcitabine or carboplatin plus paclitaxel) [5,6]. Several single chemotherapy regimens, including docetaxel, pemetrexed, erlotinib and gefitinib, have been recommended as the second-line chemotherapy for patients with advanced NSCLC progressing after the first-line treatment [7,8]. However, there is still no standard guideline for the second-line chemotherapy.

The albumin-bound nanoparticle formulation of paclitaxel (*nab*-paclitaxel) is a solvent-free option for chemotherapy. *Nab*-paclitaxel combined with carboplatin has been approved by FDA for the first-line treatment of advanced NSCLC regardless of histologic subtypes in 2012 [9,10]. A phase III trial demonstrated that *nab*-paclitaxel plus carboplatin significantly improved overall response rates compared with conventional, solvent-based paclitaxel plus carboplatin in patients with advanced NSCLC [11]. Recently, increasing evidence suggested that *nab*-paclitaxel, either as a single chemotherapy reagent or in combination with gemcitabine, can be used as the second-line therapy for improving patients’ survival rates in pancreatic cancer [12,13] or gastric cancer [14]. However, there is still insufficient evidence to prove whether *nab*-paclitaxel is effective in the second-line chemotherapy for patients with advanced NSCLC. Therefore, we evaluated the efficacy and safety of single reagent *nab*-paclitaxel as the second-line treatment for patients who have advanced in this randomized placebo-controlled phase II clinical study in China.

## Materials and methods

### Ethics statement

All protocols in the present study were conducted in accordance with the Declaration of Helsinki and Good Clinical Practice Guidelines. All procedures and treatments were reviewed and approved by the Ethics Committees at the People’s Hospital of Dongyang city. All participating patients signed the consent forms.

### Patients

Between October 2011 and October 2014, there were 92 eligible patients with advanced NSCLC participating in the present study at the People’s Hospital of Dongyang city in Dongyang, Zhejiang province, China. Patients with advanced NSCLC were randomly stratified (in 1:1 ratio) into two treatment groups, placebo-controlled group (46 patients) and the nab-paclitaxel group (46 patients). Patients were included in the study if they were between 18 and 75 years, had an Eastern Cooperative Oncology Group (ECOG) performance status (PS) between 0 and 3, had advanced (stage III/IV) NSCLC, had first-line platinum-based chemotherapy but still had progressing disease, had a life expectancy no shorter than 3 months and had adequate hepatic, renal or bone marrow functions. Patients were excluded if they were pregnant, had the radiation therapy within 1 week before enrollment, had the first-line chemotherapy within 3 weeks before enrollment, had major surgery within 6 months before enrollment or had other incurable diseases or tumours.

### Study design and treatment

This is an open-label, placebo-controlled, randomized phase II clinical study that evaluates the efficacy and safety of *nab*-paclitaxel as second-line chemotherapy for Chinese patients with advanced NSCLC after the platinum-based first-line chemotherapy failure.

Computed tomographic scans of the chest and abdomen were obtained 2 weeks before the study. At least one measurable lesion was required for patients to be evaluated for a response.

For drug administration, patients in the *nab*-paclitaxel group were intravenously administrated with 150 mg/m^2^ of *nab*-paclitaxel on days 1, 8 and 15 of a 4-week cycle. Patients should receive at least two cycles of treatment until failure to tolerate the adverse events (AEs) or request to discontinue the treatment with an upper limit of six cycles. Matched placebo was given to the patients in the control group.

Toxicities or AEs were estimated based on the guidelines of National Cancer Institute’s Common Terminology Criteria for Adverse Events version 3.0 (CTCAE v3.0). Supportive treatment for tumour-related AEs was available for all patients. If grade 3 or 4 AEs were observed, *nab*-paclitaxel dose was reduced to 100 mg/m^2^ and patients were provided with 4 mg dexamethasone plus 1 mg vitamin B_12_ to reduce toxicities.

### Statistical analysis

The primary endpoint in the present study was response rates (RRs). The secondary endpoints were overall survival (OS), progression-free survival (PFS) or AEs. OS and PFS were estimated by the Kaplan–Meier model. The comparisons of PFS and OS were conducted with a considered log-rank test with a confidence interval (CI) of 95%.

## Results

### Patients

From October 2011 to October 2014, a total of 92 patients with advanced NSCLC were eligible for the study. They were randomly assigned to placebo-controlled group (*n*=46) and *nab*-paclitaxel group (*n*=46) in a 1:1 ratio. As shown in Table 1, the percentages of male/female patients in placebo-controlled group and *nab*-paclitaxel group were 56.5%/43.5% and 52.2%/47.8% respectively. The average ages were 57.2 years (25–73 years) in placebo-controlled group and 58.5 years (23–74 years) in *nab*-paclitaxel group respectively. In both the groups, more than 90% of patients had ECOG PS between 0 and 2, more than 90% patients were current or former smokers and approximately 70% of the patients had adenocarcinoma. After first-line platinum-based chemotherapy, the percentages of patients showing responses in placebo-controlled group and *nab*-paclitaxel group were 32.6 and 30.4% respectively. Also, the percentages of patients with stable diseases in placebo-controlled group and *nab*-paclitaxel group were 50.0 and 54.3% respectively. There were no significant differences in the clinical characteristics and baseline demographics suggesting that the patients’ baseline characteristics were well balanced between the two groups.

**Table 1:** The clinical characteristics and baseline demographics of Chinese patients with advanced NSCLC

Category	Placebo (*n*=46)		*Nab*-paclitaxel (*n*=46)		*P-*value
	Number	%	Number	%	
Sex, male/female	26/20	56.5/43.5	24/22	52.2/47.8	>0.05
Age, years (range)	57.2 (25–73)		58.5 (23–74)		>0.05
**ECOG PS**					
0–1	31	67.4	29	63.0	>0.05
2	13	28.3	17	37.0	>0.05
3	2	4.3	0	0.0	>0.05
**Lung cancer subtype**					
Adenocarcinoma	32	69.6	36	78.2	>0.05
Squamous-cell carcinoma	11	23.9	8	17.4	>0.05
Others	3	6.5	2	4.3	>0.05
**Smoking history**					
Present and former	43	93.5	42	91.3	>0.05
Never	3	6.5	4	8.7	>0.05
**Response to first-line chemotherapy**					
Complete + partial response	15	32.6	14	30.4	>0.05
Stable disease	23	50.0	25	54.3	>0.05
Progressive disease	8	17.4	7	15.2	

### Efficacy

The cut-off date of the present study was October 4th 2014 and the duration of treatment was approximately 2–6 months. The RRs for patients in placebo-controlled group and *nab*-paclitaxel group are summarized in Table 2. The complete RRs were low in both groups, estimated as 0.0% (0/46) in placebo-controlled group and 2.2% (1/46) in *nab*-paclitaxel group. Encouragingly, the partial RR was much better in *nab*-paclitaxel group (17.4%, 8/46) than that in placebo-controlled group (4.3%, 2/46) (*P*<0.001). Thus, the objective RR was significantly improved, from 4.3 (2/46) in placebo-controlled group to 19.6% (9/46) in *nab*-paclitaxel group (*P*<0.001). Furthermore, the stable disease was also significantly improved, from 15.2 (7/46) in placebo-controlled group to 52.2% (24/46) in *nab*-paclitaxel group (*P*<0.001). The confirmed progressive disease rates were 67.4 (31/46) in placebo-controlled group and 43.5% (20/46) in *nab*-paclitaxel group.

**Table 2:** Efficacy of Chinese patients with advanced NSCLC

	Placebo (*n*=46)		Nab-paclitaxel (*n*=46)	
**Responses**	Number	%	Number	%
Complete response	0	0.0	1	2.2
Partial response	2	4.3	8	17.4
Objective response	2	4.3	9	19.6
Stable disease	5	10.9	15	32.6
Disease control rate	7	15.2	24	52.2
Progressive disease	31	67.4	20	43.5

**Figure 1:**
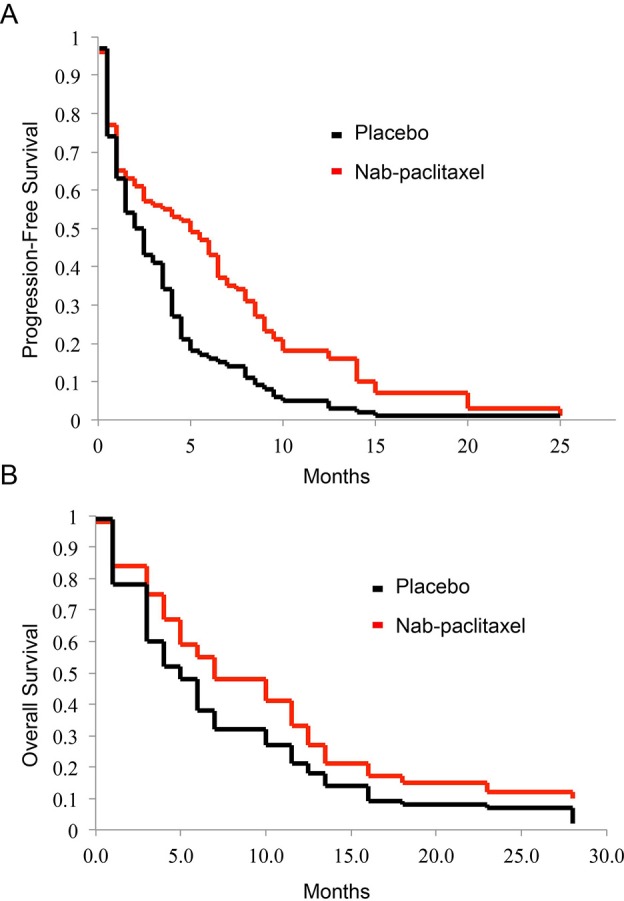
Comparison of PFS and OS for placebo-controlled and *nab*-paclitaxel groups. (**A**) PFS and (**B**) OS were estimated by Kaplan–Meier curves for patients with advanced NSCLC, in placebo-controlled group and *nab*-paclitaxel group.

The median PFS and OS were compared between the two groups (Figure 1). The PFS was 4.6 months (95% CI: 3.4–6.7 months) for *nab*-paclitaxel, compared with 2.0 months (95% CI: 0.9–4.3 months) for placebo, representing a 56% reduction in disease progression (hazard ratio: 0.62; 95% CI: 0.33-0.81; *P*<0.001). The median OS was 6.3 months (95% CI: 3.9-8.2 months) for *nab*-paclitaxel, compared with 4.9 months (95% CI: 2.1–5.9 months) for placebo, representing a 22% reduction in disease progression (hazard ratio: 0.71; 95% CI: 0.33–0.85; *P*<0.001).

### Safety

Grades 3 and 4 AEs were compared between the two groups (Table 3). The total grades 3 and 4 AE rates were 6.5 (4/46) for placebo-controlled and 34.8% (16/46) for *nab*-paclitaxel respectively. In both the groups, the highest rate of grade 3 or 4 AEs was diarrhea, accounting for 4.3 (2/46) of total patients in placebo-controlled group and 13.0% (6/46) of total patients in the nab-paclitaxel group. In placebo-controlled group, other grades 3 and 4 AEs were nausea (2.2%, 1/46), fatigue (2.2%, 1/46), thrombocytopaenia (2.2%, 1/46) and leucopenia (4.3%, 2/46). In the *nab*-paclitaxel group, other grades 3 and 4 AEs were fatigue (13.0%, 6/46), infection (10.9%, 5/46), leucopenia (10.9%, 5/46) and rash (8.7%, 4/46).

**Table 3:** Grades 3 and 4 AEs of patients with advanced NSCLC

AEs	Placebo (*n*=46)		Nab-paclitaxel (*n*=46)	
	Number	%	Number	%
Total	4	6.5	16	34.8
Rash	0	0	4	8.7
Nausea	1	2.2	3	6.5
Vomiting	0	0	2	4.3
Stomatitis	0	0	2	4.3
Pruritus	0	0	0	0
Anorexia	0	0	3	6.5
Diarrhoea	2	4.3	6	13.0
Paronychia	0	0	3	6.5
Fatigue	1	2.2	6	13.0
Infection	0	0	5	10.9
Thrombocytopaenia	1	2.2	4	8.7
Pulmonary fibrosis	0	0	2	4.3
Leucopenia	2	4.3	5	10.9

## Discussions

In the present study, we performed a randomized placebo-controlled phase II study to investigate the efficacy and safety of *nab*-paclitaxel as a single reagent to treat Chinese patients with advanced NSCLC in second-line chemotherapy. Our study showed that the objective response was significantly improved from 4.3 (2/46) for placebo to 19.6% (9/46) for *nab*-paclitaxel. We also showed that PFS and OS were significantly improved by *nab*-paclitaxel. The PFS was improved from 2.0 (95% CI: 0.9–4.3 months) in placebo-controlled group to 4.6 months (95% CI: 3.4–6.7 months) for *nab*-paclitaxel group (hazard ratio: 0.62; 95% CI: 0.33–0.81; *P*<0.001). The median OS was also improved, from 4.9 months (95% CI: 2.1–5.9 months) in placebo-controlled group to 6.3 months (95% CI: 3.9–8.2 months) in nab-paclitaxel group (hazard ratio: 0.71; 95% CI: 0.33–0.85; *P*<0.001).

Currently, three chemotherapy reagents, docetaxel, permetrexed and erlotinib have been approved by FDA to be used as second-line chemotherapy reagents for patients with NSCLC. Shepherd et al. [15] conducted a phase III, randomized, placebo-controlled, double-blind trial to determine whether erlotinib prolonged survival in NSCLC after the failure of first- or second-line chemotherapy. It showed that erlotinib (150 mg q.d.) significantly increased PFS (2.2 months compared with 1.8 months for placebo, *P*<0.001) and OS (6.7 months compared with 4.7 months for placebo, *P*<0.001) in patients with stage IIIB or IV NSCLC who failed first- or second-line treatments. Hanna et al. [16] compared the efficacy and toxicity of pemetrexed compared with docetaxel in patients with advanced NSCLC having previous chemotherapy in a phase III trial. It showed that pemetrexed and docetaxel had similar efficacy in the median PFS (2.9 months compared with 2.9 months, *P*>0.05) and median survival time (8.3 months compared with 7.9 months, *P*>0.05). Li et al. [17] investigated the effects of erlotinib and pemetrexed on the median PFS of EGFR wild-type lung adenocarcinoma patients who previously underwent one platinum-based chemotherapy. The results showed that the median PFS was 4.1 months (95% CI: 1.6–6.6 months) in the erlotinib group compared with 3.9 months (95% CI: 2.7–5.1 months) in the pemetrexed group. Hu and Zhang [18] reported that the median PFS of patients with advanced NSCLC after first-line platinum-based chemotherapy was 3.5 months (95% CI: 1.9–5.8 months) receiving *nab*-paclitaxel 100 mg/m (2) (i.v.) on days 1, 8 and 15 of a 28-day cycle. Liu et al. [19] compared the median PFS of patients with advanced NSCLC after unsuccessful first-line chemotherapy and the results showed that the *nab*-paclitaxel had better PFS (5.1 months) than pemetrexed (4.6 months). Similarly, our study also showed that the *nab*-paclitaxel had good efficacy in PFS (4.6 months). However, there were several factors in the present study contributing to this result. First, the sample size of the present study was relatively small as compared with the previous trials. A large pool of patients with multiple-centre study would yield more accurate rates on survival. Second, only Chinese patients were enrolled in the present study and the differences of ethnicity probably resulted in the differences of survival between these studies. Third, it was not a multicentre study that caused inevitable and unpredictable errors.

In the present study, we found that the patients were more likely to experience grade 3/4 AEs in nab-paclitaxel group, than in placebo-controlled group. The total incidence rate of grade 3 or 4 AEs was 34.8% for *nab*-paclitaxel. Shepherd et al. [15] reported that 26 patients (5%) discontinued treatment due to the severe toxic effects and 19% erlotinib group required dose reductions because of drug-related toxic effects. Among them, most frequent AE was rash (12%) and diarrhoea (5%). Hanna et al. [16] showed that patients receiving docetaxel were more likely to have grade 3 or 4 neutropaenia (40.2% compared with 5.3%, *P*<.001), febrile neutropaenia (12.7% compared with 1.9%, *P*<.001), neutropaenia with infections (3.3% compared with 0.0%, *P*=0.004), hospitalizations for neutropaenic fever (13.4% compared with 1.5%, *P*<.001), hospitalizations due to other drug-related AEs (10.5% compared with 6.4%, *P*=0.092) compared with patients receiving pemetrexed. Cappuzzo et al. [20] reported that most common grade 3 AEs of erlotinib treatment were rash (9%) and diarrhoea (2%). Liu et al. [19] reported that the most occurred grade 3/4 AEs were rash (*n*=4, 7.1%), nausea (*n*=2, 3.6%), paronychia (*n*=2, 3.6%), anorexia (*n*=2, 3.6%) and thrombocytopaenia (*n*=2, 3.6%) in patients with advanced NSCLC receiving pemetrexed treatment. Therefore, in the present study, the toxicity rates were similar with that of erlotinib and docetaxel, however, it seemed to be slightly higher than that of pemetrexed.

Overall, the promising results from our study showed that *nab*-paclitaxel may be used as a new chemotherapy reagent for second-line treatment for patients with advanced NSCLC.

## Abbreviations

AE: adverse event
CI: confidence interval
ECOG: Eastern Cooperative Oncology Group
NSCLC: non-small cell lung cancer
OS: overall survival
PFS: progression-free survival
PS: performance status
RR: response rate
